# Global cDNA Amplification Combined with Real-Time RT–PCR: Accurate Quantification of Multiple Human Potassium Channel Genes at the Single Cell Level

**DOI:** 10.1002/1097-0061(20000930)17:3<201::AID-YEA30>3.0.CO;2-R

**Published:** 2000

**Authors:** A. Al-Taher, A. Bashein, T. Nolan, M. Hollingsworth, G. Brady

**Affiliations:** 1 School of Biological Sciences University of Manchester Oxford Road Manchester M13 9PT UK; 2 G.38 Stopford Building School of Biological Sciences University of Manchester Oxford Road Manchester M13 9PT UK

## Abstract

We have developed a sensitive quantitative RT–PCR procedure suitable for the analysis of small samples, including single cells, and have used it to measure levels of potassium channel mRNAs in a panel of human tissues and small numbers of cells grown in culture. The method involves an initial global amplification of cDNA derived from all added polyadenylated mRNA followed by quantitative RT–PCR of individual genes using specific primers. In order to facilitate rapid and accurate processing of samples, we have adapted the approach to allow use of TaqMan^™^ real-time quantitative PCR. We demonstrate that the approach represents a major improvement over existing conventional and real-time quantitative PCR approaches, since it can be applied to samples equivalent to a single cell, is able to accurately measure expression levels equivalent to less than 1/100th copy/cell (one specific cDNA molecule present amongst 10^8^ total cDNA molecules). Furthermore, since the initial step involves a global amplification of all expressed genes, a permanent cDNA archive is generated from each sample, which can be regenerated indefinitely for further expression analysis.


**Yeast**
*Yeast* 2000; **17**: 201–210.

Research Article

### Global cDNA amplification combined with real-time RT–PCR: accurate quantification of multiple human potassium channel genes at the single cell level

A. Al-Taher, A. Bashein, T. Nolan, M. Hollingsworth and G. Brady*
*School of Biological Sciences, G.38 Stopford Building, University of Manchester, Oxford Road, Manchester M13 9PT, UK*

*Correspondence to:
G. Brady, G.38 Stopford Building, School of Biological Sciences, University of Manchester, Oxford Road, Manchester M13 9PT, UK.
E-mail: Ged.Brady@man.ac.uk

Received: 23 June 2000
Accepted: 19 July 2000

### Abstract

**We have developed a sensitive quantitative RT–PCR procedure suitable for the analysis of small samples, including single cells, and have used it to measure levels of potassium channel mRNAs in a panel of human tissues and small numbers of cells grown in culture. The method involves an initial global amplification of cDNA derived from all added polyadenylated mRNA followed by quantitative RT–PCR of individual genes using specific primers. In order to facilitate rapid and accurate processing of samples, we have adapted the approach to allow use of TaqMan™ real-time quantitative PCR. We demonstrate that the approach represents a major improvement over existing conventional and real-time quantitative PCR approaches, since it can be applied to samples equivalent to a single cell, is able to accurately measure expression levels equivalent to less than 1/100th copy/cell (one specific cDNA molecule present amongst $10^8$ total cDNA molecules). Furthermore, since the initial step involves a global amplification of all expressed genes, a permanent cDNA archive is generated from each sample, which can be regenerated indefinitely for further expression analysis. Copyright © 2000 John Wiley & Sons, Ltd.**

**Keywords:** RT–PCR; single-cell; quantitative; real-time PCR

### Introduction

Potassium channels are a large family of ion channels that are present in essentially all cell types and mediate a wide range of physiological functions. Physiological, pharmacological and expression studies have demonstrated that the behaviour of a variety of tissues is influenced by the pattern of potassium channels present. For example, the large conductance calcium-dependent potassium channel $BK_{Ca}$ (also known as Maxi-K or slo channel) plays a key role in a range of cellular functions, including neuronal spike shaping and neurotransmitter release (Kaczorowski *et al*., 1996), setting contractile tone (Nelson *et al*., 1995), modulating myometrial activity (Anwer *et al*., 1992, 1993) and electrical tuning of cochlear hair cells (Art *et al*., 1995; Ramanathan *et al*., 1999). With the overall aim of examining mRNA levels of a panel of potassium channels in human tissue samples, we set out to develop an approach with the following characteristics. First, the method should provide a means of determining the approximate number of mRNA molecules/cell in each sample studied, thereby establishing the likely physiological consequence of the detected expression. Second, the method should be capable of detecting a wide range of expression levels, including those less than one part in a million (equivalent to less than one copy/cell). Third, it should be applicable to small samples, including single cells, making it possible to correlate physiological measurements using patch-clamping with expression profiles. Fourth, the method should allow the examination of multiple genes from the same sample in order to establish the presence and level of a range of potassium channels and their subunits. Finally, the method should ideally allow retrospective screening of accumulated samples in order to facilitate analysis of novel potassium channels.

Copyright © 2000 John Wiley & Sons, Ltd.

Clearly, classical techniques for determining mRNA levels, such as Northern blot analysis and RNase protection assays, are not capable of fulfilling these criteria, since they require large amounts of total RNA. With the advent of the polymerase chain reaction (PCR) and its application to the analysis of mRNA [reverse transcriptase PCR (RT–PCR)], it is now possible to analyse mRNA levels in small samples including individual cells (Brady *et al.*, 1990; Brady and Iscove, 1993; Rappolee *et al.*, 1989). Although RT–PCR can be used to quantify expression, existing methods are generally time-consuming, restricted to a small number of target genes and not suitable for the examination of samples as small as a single cell. To overcome this limitation we have developed an approach based on an initial global amplification of cDNA copies of all polyadenylated mRNAs followed by real-time quantitative PCR directed towards specific genes. The resultant approach exhibits the precision associated with real-time PCR (Preudhomme *et al.*, 1999; Steuerwald *et al.*, 1999; Wang and Brown, 1999) and in addition has several valuable features which are not part of current real-time or standard quantitative techniques. These features include the ability to analyse samples as small as a single cell and the ability to determine absolute expression levels as low as one specific target cDNA in amongst $10^8$ total cDNAs. Furthermore, since the initial step is a global amplification, the method generates a renewable archive of representative cDNAs, which can also be used for retrospective screening of accumulated samples. To validate the approach we have examined the $IK_{Ca}$ potassium channel, which is highly expressed in placenta (Ishii *et al.*, 1997; Logsdon *et al.*, 1997) and $BK_{Ca}$ ($\alpha$- and $\beta$ subunits) which is both physiologically active (Khan *et al.*, 1993) and detectable at the level of protein and mRNA (Song *et al.*, 1999) in uterus.

### Materials and methods

### Global Amplification of cDNA copies of all polyadenylated mRNAs (PolyAPCR)

Tissue RNAs were obtained from commercial suppliers (human lung, heart, placenta and breast from Ambion, and remaining tissue RNAs from Clontech). PolyAPCR was carried out essentially as previously described (Brady and Iscove, 1993). In brief, for each sample 0.5 µg total RNA in a volume of 0.5 µl was added to 10 µl first lyse buffer [600 U/ml Prime RNase Inhibitor (5′–3′ incl.), 50 mM Tris–HCl (pH 8.3), 75 mM KCl, 3 mM $MgCl_2$, 0.5% Nonidet P-40, 10 µm dNTPs, 23 nM dT24 oligo], incubated at 65°C for 1 min, allowed to cool to 22°C (room temperature) and transferred to wet ice. After adding 12.5 units AMV reverse transcriptase (Boehringer-Mannheim), the reverse transcriptase reaction was initiated by transferring the samples from ice to 37°C. After 15 min incubation at 37°C the reverse transcriptase was inactivated by heating to 65°C for 10 min and samples were returned to ice. For the addition of the 5′ priming site, a terminal transferase reaction was carried out by adding 10 µl 2× tailing buffer [200 mM potassium cacodylate (pH 7.2), 4 mM $CoCl_2$, 0.4 mM DTT, 1 mM dATP] containing 10 U terminal deoxynucleotide transferase (Gibco BRL) and incubating the samples at 37°C for 15 min followed by heat inactivation at 65°C for 10 min. Following return of the samples to wet ice, 40 µl PCR mix [23.5 mM Tris–HCl (pH 8.3), 117.4 mM KCl, 8.2 mM $MgCl_2$, 2.2 mM dNTPs, 0.23% Triton X-100, 47 µg/ml BSA, 85 units/ml Taq polymerase, 8.33 µm oligo Not140] was added to all samples and PCR was performed using two linked sets of 25 cycles. The first 25 cycles were: denaturation at 94°C for 1 min, primer annealing at 42°C for 2 min and extension at 72°C for 6 min; and the second 25 cycles involved denaturation at 94°C for 1 min, primer annealing at 42°C for 1 min and extension at 72°C for 2 min. Following evaluation of the PolyAPCR products (PolyAcDNA) by agarose gel electrophoresis and densitometry, all samples were adjusted to a final concentration of 0.5 µg/ml.

### Gene-specific PCR

For each gene studied, PCR primer pairs directed towards the mRNA sequence present within 300 bases of the polyA addition site were designed as previously described (Núnêz *et al.*, 2000). Sequences for PCR primers presented in the 5′–3′ orientation, together with the Accession Nos of the target genes, are listed in Table 1. PCR reactions were carried out in a 22 µl reaction volume containing 1 ng PolyAcDNA, 0.33 µm each oligonucleotide, 0.5 units TaKaRa Ex Taq (TaKaRa), 0.25 mM dNTPs in buffer supplied by the manufacturer. PCR was performed without mineral oil in a Techne PHC-3

Copyright © 2000 John Wiley & Sons, Ltd.

Table 1. Conventional primers and probes

| Name | Oligonucleotides | Supplier |
|---|---|---|
| Human GAP (X01677) | | |
| HGAP-5p | CCA GCA AGA GCA CAA GAG GAA GAG | Genosys |
| HGAP-3p | AGC ACA GGG ATA CTT TAT TAG ATG | Genosys |
| HGAP probe | CAC ACT CAG ACC CCC ACC ACA C | Genosys |
| Human $BK_{Ca}\alpha$ | | |
| BKCa A 5p | CCC CTA GTA TTG GAT CAT GAA GAG C | Genosys |
| BKCa A 3p | CAG TGG CTA GGT CAT GCA GAA C | Genosys |
| BKCa A probe | GTG CCA GTT AAA GTG CTC AAC AGG | Genosys |
| Human $BK_{Ca}\beta$ (AA412682) | | |
| BKCabeta-5p | CAT AAG GCC AGC CAG TTC TAG CTC | Genosys |
| BKCabeta-3p | GTG TTC TAG AAA TGG GAG CTG CTG | Genosys |
| BKCabeta probe | GTT GTC TCA AAG CGG TAC CCA TCC | Genosys |
| Human $IK_{Ca}$ (AF022797) | | |
| Ikca-5p | CTC TCA GTT ACA AGT GCA GGC GAC | Genosys |
| Ikca-3p | AGA GCA GAG GCT GGT GAG TTA CAC | Genosys |
| Ikca-probe | AGC TGA AGA ACT GGG TAT GAG GC | Genosys |

All oligonucleotides are based on public domain sequences (Accession Nos indicated in parentheses), with the exception of $BK_{Ca}\alpha$ which was based on sequences kindly provided by Dr Martin Wallner. For all oligonucleotides 5p indicates denotes a forward primer, 3p denotes a reverse primer and probe denotes an internal probe primer.

thermocycler fitted with a heated lid by heating the samples for 5 min at 95°C, followed by 20–30 cycles of 30 s at 94°C, 30 s at 56°C and 1 min at 72°C.

### Preparation of gene-specific quantity standard (GSQS)

Following successful gene-specific amplification, PCR products were purified using GFX PCR DNA and Gel Band purification Kits (Pharmacia Biotech) as recommended by the manufacturer. Purified PCR products were resolved by agarose gel electrophoresis, visualized by ethidium bromide staining and the concentration of each band calculated by densitometric analysis of the gel images using a GS-700 Imaging Densitometer (Bio-Rad) and Molecular Analyst software, Version 1.5 (Bio-Rad). A gene-specific quantity standard (GSQS) for each PCR product was generated by using the purified bands to create a dilution series, where the number of DNA molecules ranged from $1.5\times10^{10}$ to $1.5\times10^{4}$ /ml (Núnêz *et al.*, 2000). For example, the 180 bp human glyceraldehyde-3-phosphate dehydrogenase (GAP) fragment was first diluted to 2.97 ng/ml ($1.5\times10^{10}$ molecules/ml) and from this solution serial 10-fold dilutions were prepared in TE (10 mM Tris–HCl, pH 8.0, 1 mM EDTA) containing 0.5 μ/ml sonicated carrier λDNA.

### Hybridization analysis of PCR products

For each gene, PCR (20–35 cycles) was applied to 1 ng PolyAcDNA (equivalent to $3\times10^{9}$ total cDNA molecules based on an average molecular size of 300 bases) from each sample and 2 μl from six relevant gene-specific quantity standards equivalent to a range of $3\times10^{7}$–30 molecules. PCR products were resolved by agarose gel electrophoresis, visualized by ethidium bromide staining, photographed and nylon filters prepared as previously described (Brady *et al.*, 1995). For each gene an internal oligonucletide probe (Table 2) was end-labelled with fluorescent dUTP (Amersham) using terminal transferase (Gibco Life Sciences) and hybridized to the relevant filter at 45°C for 16 h in 5×SSC, 0.1% SDS, 1:20 blocking agent (Amersham), 5% dextran sulphate. Following the removal of excess probe, filters were washed 2×30 minutes in 0.5 SSC+0.1% SDS and signals detected using a Gene Images CDP-Star detection module (Amersham Life Sciences) as described by the manufacturer. For each gene, separate PCR reactions were carried out for 20, 25, 23 and 35 cycles, all products hybridized as described above, and the resultant autoradiographs quantified by densitometric analysis using a GS-700 Imaging Densitometer (Bio-Rad) and Molecular Analyst software, Version 1.5 (Bio-Rad).

Copyright © 2000 John Wiley & Sons, Ltd.

Table 2. Taqman primers and probes

| Name | Oligonucleotides | Supplier |
|---|---|---|
| Human GAP (X01677) | | |
| HUMANGAP-55F | ACA CTC AGA CCC CCA CCA CA | MWG |
| HUMANGAP-133R | CAT AGG CCC CTC CCC TCT T | MWG |
| HUMANGAP-80T | TCT CCC CTC CTC ACA GTT GCC ATG TAG A* | MWG |
| Human $BK_{Ca}\alpha$ | | |
| BKCAA-58F | AGT TAC GCC CTC AGA ACA TTT ATA ACT A | MWG |
| BKCAA-157R | AGT TAC GCC CTC AGA ACA TTT ATA ACT A | MWG |
| BKCAA-87T | AAG CTA TGT GAG TTT TAC AAT GCT TTT AA* | MWG* |
| Human $BK_{Ca}\beta$ (AA412682) | | |
| BKCABETA-29F | CCC TGT TGT CTC AAA GCG GTA | MWG |
| BKCABETA-129R | TGG GAG CTG CTG TTG CTC TTA | MWG |
| BKCABETA-74T | CCA GCG AGC CTC CCT TCT TTG TTT GA* | MWG* |
| Human $IK_{Ca}$ (AF022797) | | |
| Ikca-F | CAG GAT TCT GGG AGG CTT CA | PE ABI |
| Ikca-R | CGC CCC AGC CTC ATA CC | PE ABI |
| Ikca-T | TTA CCG CTG GCC GAG CTG AAG AAC T* | PE ABI |

*Oligonucleotides modified by the addition of FAM (5′)and TAMRA (3′)
All oligonucleotides are based on public domain sequences (Accession Nos indicated in parentheses) with the exception of $BK_{Ca}\alpha$, which was based on sequences kindly provided by Dr Martin Wallner. For all oligonucleotides F indicates denotes a forward primer, R denotes a reverse primer and T denotes an internal fluorescent probe primer.

### Preparation of polyAcDNAs from 10- and one-cell dilutions

One thousand freshly growing human lymphoblastoid CCRF CEM C7A cells (Norman and Thompson, 1977) were directly lysed in 100 µl first lyse buffer and two serial 10-fold dilutions carried out to yield cell lysates at concentrations of 100 and 10 cells/ml. To establish that global amplification is feasible at the level of 10 and one cell(s), three independent PolyAPCR reactions were prepared from 10 µl aliquots taken from the 100 and 10 cell/ml cell lysates. Following PolyAPCR, the resultant PolyAcDNA*s* from both 10 and one cell triplicates were analysed as described below.

### TaqMan® real-time quantitative PCR

TaqMan® (PE Applied Biosystems) PCR primers and probes were designed for each gene using Primer Express® and are listed in Table 2. Primers were supplied by either PE Applied Biosystems (PE ABI), Genosys, or MWG and fluorescently labelled probes [5′ 6-carboxyfluorescein (FAM); 3′ 6-carboxytetramethylrhodamine (TAMRA)] were supplied by either MWG or PE ABI (see Table 2). For each gene, TaqMan® PCR was applied to 1 ng PolyAcDNA (equivalent to $3\times10^9$ total cDNA molecules) from each tissue and 2 µl of all six relevant gene-specific quantity standards (equivalent to range of $3\times10^7$/300 molecules) using a TaqMan® Gold kit, as recommended by the manufacturer. Samples were analysed using an ABI Prism 7700 Sequence Detection System (PE Applied Biosystems) as recommended by the manufacturer.

### Results

### Global amplification and gene-specific PCR reveals a striking tissue-specific pattern for potassium channel genes

Agarose gel electrophoresis and ethidium bromide staining was used to detect the amplification products from 25 cycles of gene-specific PCR applied to PolyAcDNA*s* derived from human tissues and a $BK_{Ca}\alpha$ serial standard (Figure 1A). Based on the appearance of DNA bands in the first five $BK_{Ca}\alpha$ serial standards (Figure 1A), the threshold of PCR detection using agarose gel electrophoresis/ethidium bromide staining for these conditions was around $3\times10^3$ target molecules present at the start of the PCR reaction. Of the six tissue PolyAcDNA*s* examined, a strong band was detected in samples from uterus that was similar to, or brighter than, the band seen in serial standard

Copyright © 2000 John Wiley & Sons, Ltd. 

containing $3 \times 10^5$ $BK_{Ca}\alpha$ cDNA molecules. Faint but reproducible bands were also seen in the breast and placenta lanes (Figure 1A).

To increase the sensitivity and specificity of PCR detection, internal oligonucleotide probes were used to detect gene-specific PCR products (Figure 1B). As can be seen from comparing the $BK_{Ca}\alpha$ bands in Figures 1A and 1B, detection of PCR products by Southern hybridization increased the detection sensitivity approximately 100-fold and revealed low-level expression of $BK_{Ca}\alpha$ in lung and heart, whereas expression in foetal liver remained undetectable. Since annealing temperature, number of cycles and primer concentration were identical for both tissue and serial standard samples, an estimate of the abundance of $BK_{Ca}\alpha$ transcripts in the starting tissue samples can be made by comparing amplification products derived from the tissue PolyAcDNA*s* to the $BK_{Ca}\alpha$ serial standards. For example, band intensities in both Figure 1A and 1B indicate that the$BK_{Ca}\alpha$ PCR product from uterus is equivalent to at least $3 \times 10^5$ $BK_{Ca}\alpha$ cDNA molecules present at the beginning of the PCR reaction. From a visual comparison of the bands present in the tissue samples and serial standard in Figure 1B, it can be estimated that there are around $3 \times 10^3$ $BK_{Ca}\alpha$ cDNA molecules present in the breast and placenta samples, 300 molecules present in the heart and lung samples and undetectable levels (fewer than 30 copies) in the foetal liver sample. Since 1 ng PolyAcDNA is equivalent to $3 \times 10^9$ molecules (calculation based on 300 bp as the average size of PolyAcDNA, 1 bp = 650 Da and Avagadro's number = $6 \times 10^{23}$) the abundance of $BK_{Ca}\alpha$ subunits molecules in the uterus sample can be expressed as $>1$ $BK_{Ca}\alpha$ molecule/$10^4$ total cDNA molecules. Based on the assumption that a single animal cell expresses in the order of $5 \times 10^5$ mRNA

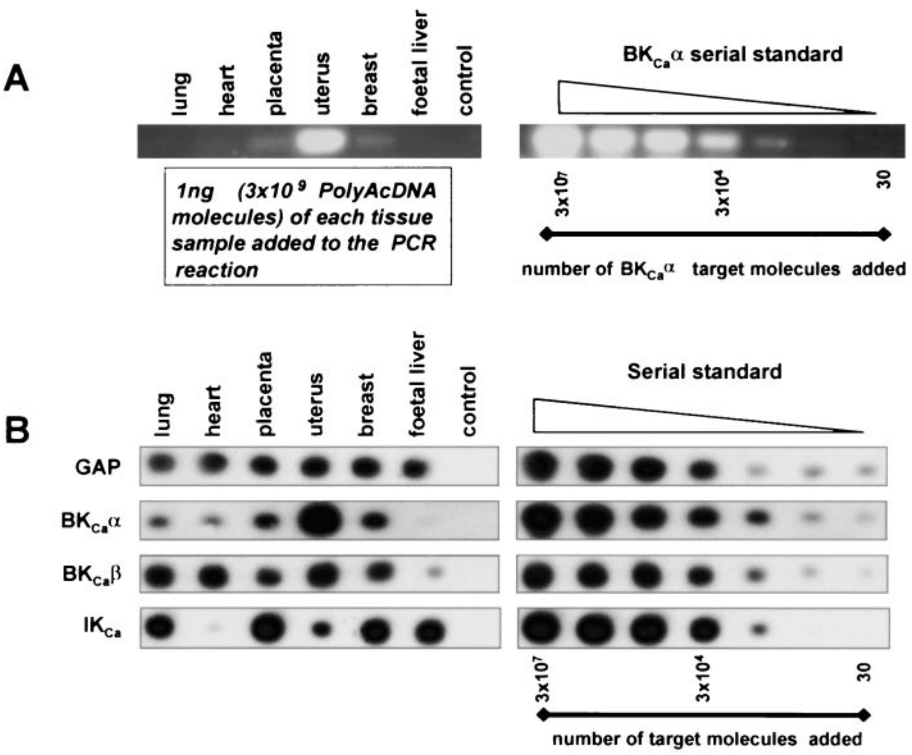

**Figure 1.** RT–PCR analysis of tissue mRNAs. (A) Results of PCR using $BK_{Ca}\alpha$ primers applied to 1 ng of tissue-derived PolyAcDNA*s*, a control sample in which no RNA was added to the initial reverse-transcriptase reaction and the $BK_{Ca}\alpha$ serial standards. PCR products have been visualized by agarose gel electrophoresis, ethidium bromide staining and UV illumination. (B) Results of PCR using the indicated primers applied to 1 ng of tissue-derived PolyAcDNAs, control and the relevant serial standards. The PCR products were detected using labelled internal oligonucleotides, as described in Materials and methods. For both panels the numbers below the serial standards refer to the number of specific target cDNA molecules present at the beginning of the PCR reaction

Copyright © 2000 John Wiley & Sons, Ltd. 

molecules (Alberts *et al.*, 1997), the average expression levels for $BK_{Ca}$ $\alpha$ in the uterus would be in the order of 50 or more mRNA molecules/cell. Based on the same assumptions, expression levels for $BK_{Ca}$ $\alpha$ for the breast and placenta samples would be in the order of 0.5 molecules/cell, for the heart and lung samples an average of 0.05 molecules/cell and for foetal liver less than 0.005 copies/cell. As expected, the housekeeping gene GAP was expressed at a level of at least 5 copies/cell in all samples, whereas the potassium channel $IK_{Ca}$ showed a restricted tissue-specific expression pattern which was distinct from the pattern for $BK_{Ca}$ $\alpha$ (Figure 1B).

### Quantitative measurement of PCR products

Although data presented in Figure 1A, B can be used to provide useful estimations of expression levels, it is also clear from an examination of the serial standard that there is a 'plateau' effect which is effectively limiting PCR product in samples which contained $3 \times 10^5$ or more target molecules. In order to improve the precision of measurements using this system, two parallel approaches were compared. In the first approach, following gene-specific PCR and Southern hybridization (Figure 1B), abundance was estimated by densitometric analysis (Figure 2C). In the second approach, TaqMan® real-time PCR was applied to both the serial standards and the tissue PolyAcDNA*s* analysed in Figure 1B. Figure 2A and B shows the results of TaqMan® analysis applied to $IK_{Ca}$ serial standards and Figure 2C shows the standardization curve generated from densitometric analysis. A comparison of TaqMan® and densitometric analysis applied to samples shown in Figure 2D revealed that, although both approaches gave comparable results, real-time PCR was able to detect low levels of $IK_{Ca}$ expression in heart which were not detected by densitometric analysis of the hybridized PCR products.

### Real-time quantitative analysis of PolyAcDNA at the single cell level

Since PolyAPCR has been widely used for global amplification of all expressed sequences from single cells (Berardi *et al.*, 1995; Brady *et al.*, 1990, 1995; Dulac and Axel, 1995), we next examined whether the current quantitative adaptation could be used at the single cell level. Previous RT–PCR analysis of individual cells has shown that for all genes

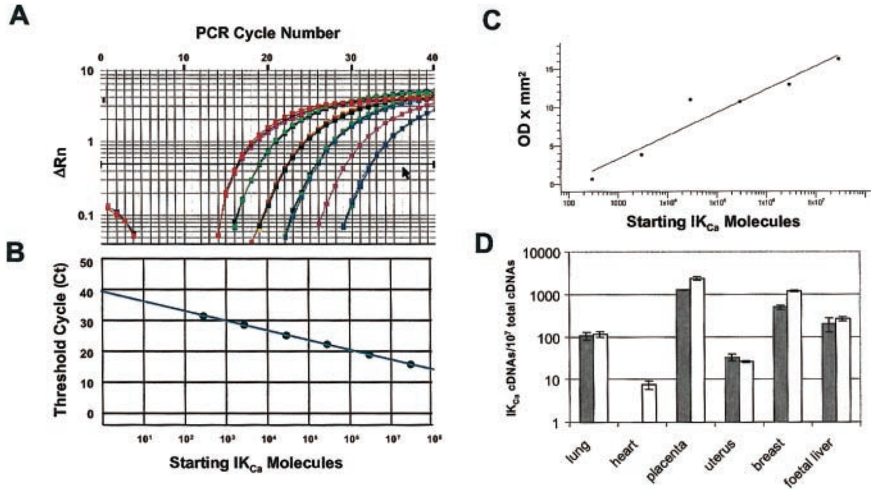

**Figure 2.** A comparison of densitometric and TaqMan quantitative RT–PCR. (A) Result of duplicate TaqMan® analysis of six $IK_{Ca}$ serial standards of $3 \times 10^7$, $3 \times 10^6$, $3 \times 10^5$, $3 \times 10^4$, $3 \times 10^3$ and 300 starting molecules (shown in the figure from left to right). Individual curves in (A) show the amount of fluorescence detected (ΔRn) for each sample in every PCR cycle and reflects the amount specific hybridization of the Taqman® to PCR product. (B) Calibration curve based on the data presented in (A), where the threshold cycle (Ct) has been calculated for each sample as the number of PCR cycles at which the ΔRn value reaches a threshold level (indicated by a black arrow in A). (C) Calibration curve based on densitometric analysis of Southern hybridization of $IK_{Ca}$ serial standards. (D) Comparison of TaqMan® (open columns) and densitometric quantitation (filled columns) of $IK_{Ca}$ mRNA levels in a panel of six tissues. For both measurements the levels of $IK_{Ca}$ expression is shown as the number of $IK_{Ca}$ cDNA molecules present in $10^7$ total cDNA molecules

Copyright © 2000 John Wiley & Sons, Ltd. 

examined there is variation in the expression levels between samples (Brady *et al.*, 1990, 1995; Brail *et al.*, 1999). In order to examine variation due simply to the method, PolyAPCR was carried out in triplicate on dilutions of a fresh cell lysate prepared from 1000 human T lymphoma cells (for details, see Materials and methods), thereby avoiding the inherent differences in gene expression exhibited by individual cells. Following triplicate reverse transcriptase, tailing and PolyAPCR applied to serial dilutions equivalent to 10 and 1 cell(s), the level of the housekeeping genes GAP, lactate dehydrogenase A (LDHA) and initiation factor 2B (IF2B) were established using TaqMan$^{TM}$ PCR (Figure 3). As can be seen from Figure 3, high levels of GAP and approximately 100-fold lower levels of LDHA and IF2B were detected in each of the 10- and 1-cell PolyAcDNA samples. Variation between samples was more apparent at the 1-cell level, particularly in the case of LDHA. Despite the variation between samples, the average expression for all genes was similar for both the 10-cell and 1-cell series (Figure 3). These results demonstrate that the approach can be applied to single cells and is sensitive enough to reliably detect genes expressed at low levels.

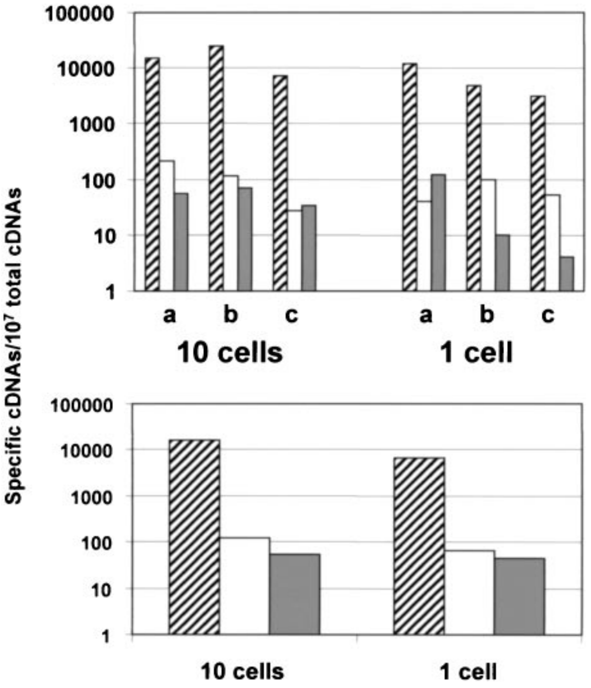

**Figure 3.** Global PolyAPCR and TaqMan quantitative RT–PCR applied at the single cell level. The top panel shows the analysis of six separate PolyAcDNAs prepared from fresh cell lysates. Three replicate PolyAcDNAs were prepared from dilutions equivalent to 10 cells (labelled 10 cells, a, b and c) and three from dilutions equivalent to 1 cell (labelled 1 cell, a, b and c). For each PolyAcDNA TaqMan quantitative RT–PCR was used to assess the level of the three housekeeping genes GAP (hatched columns), LDHA (open columns) and IF2B (filled columns). The bottom panel shows the average expression levels of GAP (hatched columns), LDHA (open columns) and IF2B (filled columns) taken from on the data shown in the top panel

### $BK_{Ca}$ $\alpha$ and $\beta$ mRNAs are highly strongly expressed in human uterus

Having established that the TaqMan$^{TM}$ approach is suitable for PolyAcDNA samples, we next used the approach to examine the levels of $BK_{Ca}\alpha$, $BK_{Ca}\beta$ and $IK_{Ca}$ mRNAs in a panel of human tissues. The results presented in Figure 4 show that the method can be used to detect and quantify levels of expression equivalent to one specific cDNA molecule in amongst $10^8$ total cDNAs. For all samples, high levels of the housekeeping gene GAP and varying levels of the potassium channels were detected. In the case of individual genes, expression levels varying over four orders of magnitude could be accurately assessed (cf. $IK_{Ca}$ expression in the adult liver and placenta samples in Figure 4). $IK_{Ca}$ levels were found to be highest in placenta and both $BK_{Ca}$ $\alpha$ and $\beta$ sub-units were highest in the uterus.

### Discussion

The results presented in Figure 4 demonstrate that the approach described can be used to identify patterns of potassium channel expression that reflect the cellular composition and physiological status of target tissues. For example, high levels of $BK_{Ca}\alpha$ and $BK_{Ca}\beta$ have been previously reported in uterus and ascribed to the preponderance of smooth muscle cells in this tissue (Khan *et al.*, 1993, 1997; Song M. 1999; Wallner *et al.*, 1995). Our finding that, among the 16 tissues examined, $BK_{Ca}$ mRNA levels (both $\alpha$ and $\beta$) were highest in the uterus sample and lowest (equivalent to $<0.1$ copy/cell) in tissues such as adult and foetal liver samples (Figure 4) are consistent with the proposed association of $BK_{Ca}$ expression and smooth muscle cells. Intriguingly, although the $BK_{Ca}\beta$ subunit is thought to act solely through moderating the activity of the $BK_{Ca}\alpha$ subunit (Kaczorowski *et al.*, 1996; Rama-

Copyright © 2000 John Wiley & Sons, Ltd. 

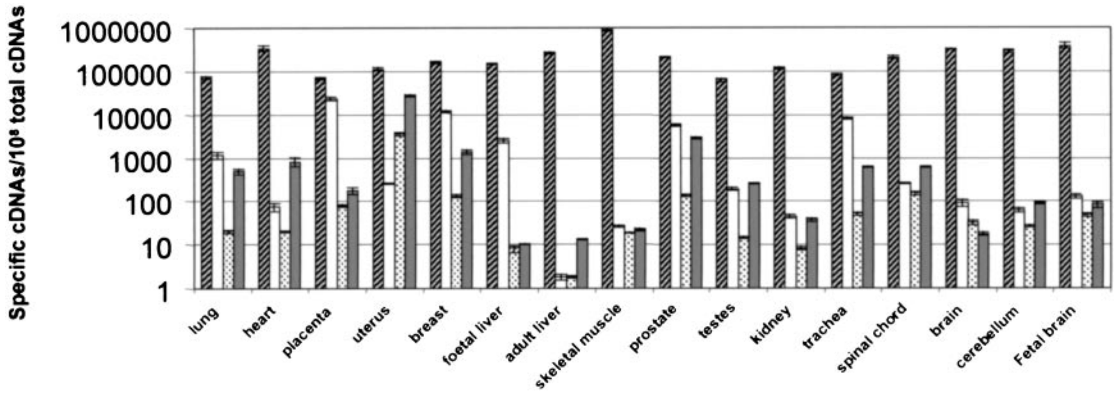

**Figure 4.** Global PolyAPCR and TaqMan quantitative RT–PCR applied to a panel of human tissue RNAs. TaqMan quantitative RT–PCR was used to assess the level of GAP (diagonal hatching), $IK_{Ca}$ (open columns), $BK_{Ca}\alpha$ (stippled) and $BK_{Ca}\beta$ (solid) in the indicated human tissues. All measurements are shown as the number of specific cDNA molecules present in $10^8$ total cDNA molecules nathan *et al.*, 1999; Tseng-Crank *et al.*, 1996), the ratio of $BK_{Ca}\alpha$ to $BK_{Ca}\beta$ varied considerably between tissue samples, with similar levels of both subunits detectable in the three brain tissues compared to 40-fold higher levels of $BK_{Ca}\beta$ than $BK_{Ca}\alpha$ detectable in the heart sample (Figure 4). Furthermore, in the case of both the heart and lung samples, expression of $BK_{Ca}\beta$ is equivalent to around 5–10 copies/cell, whereas the level of $BK_{Ca}\alpha$ in the same samples is far below one copy/cell, indicating that at least some cells in those tissues may express $BK_{Ca}\beta$ in the absence of $BK_{Ca}\alpha$. Expression of $BK_{Ca}\beta$ in the absence of $BK_{Ca}\alpha$ would suggest either that $BK_{Ca}\beta$ is able to act alone or that it is able to interact with other potassium channels. These results highlight the importance of being able to determine both absolute and relative mRNA levels for multiple genes in the same sample.

In the case of $IK_{Ca}$, 12 of the human tissues analysed in Figure 4 were previously independently analysed by two research groups using polyA mRNA and Northern analysis (Ishii *et al.*, 1997; Logsdon *et al.*, 1997). A comparison of the $IK_{Ca}$ Northern data (Ishii *et al.*, 1997; Logsdon *et al.*, 1997) and the TaqMan data in Figure 4 revealed that both *PolyAmRNA* Northern and the PolyAPCR TaqMan® identified highest levels of expression in placenta followed by medium to high expression in prostate and lung. Furthermore, the PolyAPCR TaqMan® method was able to detect low to moderate expression levels in several of tissues such as uterus, testes and spinal cord (Figure 4), which were undetectable using Northern analysis (Ishii *et al.*, 1997; Logsdon *et al.*, 1997). These results indicate that the detection limit of *PolyAmRNA* and Northern analysis used by both groups is $>1:10^6$, which is 100-fold lower than the PolyAPCR TaqMan® method described here.

PolyAPCR has been used to examine mRNA levels and patterns in a wide variety of cells including highly purified haemopoietic cell subpopulations (Cumano *et al.*, 1992; Sauvageau *et al.*, 1994), oocytes, eggs, and pre-implantation embryos from fertilization to the blastocyst stage (Rambhatla *et al.*, 1995), micro-well cultures of cells (Yan *et al.*, 1993), haemopoietic colonies (Brady *et al.*, 1990; Cheng *et al.*, 1996) and single cells (Brady *et al.*, 1990; Brady *et al.*, 1995; Dulac and Axel, 1995; Trumper *et al.*, 1993). However these studies have been based largely on filter hybridization which at very best is limited to detecting 1 specific molecule present in amongst 10,000–100,000 total cDNAs (Brady *et al.*, 1990; Cheng *et al.*, 1996; Rambhatla *et al.*, 1995). Here we demonstrate detection of levels of expression as low as one specific molecule present in amongst $10^8$ total cDNAs (Figure 4), thereby improving the overall sensitivity at least 1000-fold. Based on the assumption that a single cell contains $10^5$–$10^6$ mRNA molecules (Alberts *et al.*, 1997), we are able to detect levels of expression equivalent to 1/100th of a copy per cell. Furthermore, unlike existing TaqMan® methods (Steuerwald *et al.*, 1999; Wang and Brown, 1999) PolyAPCR TaqMan® can be applied to an unlimited number of genes from small

Copyright © 2000 John Wiley & Sons, Ltd.

samples, including single cells (Figures 3 and 4). It must be stressed that the PolyAPCR TaqMan® method is not intended as a direct alternative to mass expression screening methods such as high density cDNA array hybridization (Duggan *et al.*, 1999; Hauser *et al.*, 1998) since, at present, it is suitable for studying 10 s to 100 s of individual genes. However, PolyAPCR TaqMan® can be applied to samples several orders of magnitude smaller than those suitable for array hybridization and is able to detect expression levels at least 1000-fold lower than currently detected by array methods. In other words, unlike PolyAPCR TaqMan®, current high density cDNA array hybridization methods are unsuitable for the analysis of samples less than 1000 cells and are unable to reliably detect mammalian genes expressed either at a level lower than a few copies per cell (Duggan *et al.*, 1999). PolyAPCR TaqMan® therefore extends and complements existing mRNA profiling methods, since it can be used to provide sensitive and quantitative analysis of individual genes identified by high-density screening.

### Acknowledgements

We would like to thank Arthur Weston, Gillian Edwards, Neil Anderson, Phil Hedge and David Lydall for helpful discussions and Martin Wallner for kindly providing the full $BK_{Ca}\alpha$ DNA sequence. G.B. and T.N. were funded by AstraZeneca. A.A.-T. was funded by the King Faisal University, Saudi Arabia.

### References

Alberts B, Bray D, Lewis J, *et al.* 1994. *Molecular Biology of the Cell.* 3rd ed, New York, Garland.

Anwer K, Oberti C, Perez GJ, *et al.* 1993. Calcium-activated K+ channels as modulators of human myometrial contractile activity. *Am J Physiol* **265**: C976–985.

Anwer K, Toro L, Oberti C, Stefani E, Sanborn BM. 1992. Ca(2+)-activated K+ channels in pregnant rat myometrium: modulation by a $\beta$-adrenergic agent. *Am J Physiol* **263**: C1049–1056.

Art JJ, Wu YC, Fettiplace R. 1995. The calcium-activated potassium channels of turtle hair-cells. *J Gen Physiol* **105**: 49–72.

Berardi AC, Wang A, Levine JD, Lopez P, Scadden DT. 1995. Functional isolation and characterization of human hematopoietic stem cells. *Science* **267**: 104–108.

Brady G, Barbara M, Iscove NN. 1990. Representative *in vitro* cDNA amplification from individual hemopoietic cells and colonies. *Methods Mol Cell Biol* **2**: 17–25.

Brady G, Billia F, Knox J, *et al.* 1995. Analysis of gene-expression in a complex differentiation hierarchy by global amplification of cDNA from single cells. *Curr Biol* **5**: 909–922.

Brady G, Iscove NN. 1993. Construction of cDNA libraries from single cells. *Methods Enzymol* **225**: 611–623.

Brail LH, Jang A, Billia F, *et al.* 1999. Gene expression in individual cells: analysis using global single cell reverse transcription polymerase chain reaction (GSC RT–PCR). *Mutat Res* **406**: 45–54.

Cheng T, Shen H, Giokas D, *et al.* 1996. Temporal mapping of gene expression levels during the differentiation of individual primary hematopoietic cells. *Proc Natl Acad Sci USA* **93**: 13158–13163.

Cumano A, Paige CJ, Iscove NN, Brady G. 1992. Bipotential precursors of B cells and macrophages in murine fetal liver. *Nature* **356**: 612–615.

Duggan DJ, Bittner M, Chen YD, Meltzer P, Trent JM. 1999. Expression profiling using cDNA microarrays. *Nature Genet* **21**: 10–14.

Dulac C, Axel R. 1995. A novel family of genes encoding putative pheromone receptors in mammals. *Cell* **83**: 195–206.

Hauser NC, Vingron M, Scheideler M, *et al.* 1998. Transcriptional profiling on all open reading frames of *Saccharomyces cerevisiae*. *Yeast* **14**: 1209–1221.

Ishii TM, Silvia C, Hirschberg B, *et al.* 1997. A human intermediate conductance calcium-activated potassium channel. *Proc Natl Acad Sci USA* **94**: 11651–11656.

Kaczorowski GJ, Knaus HG, Leonard RJ, McManus OB, Garcia ML. 1996. High-conductance calcium-activated potassium channels; structure, pharmacology and function. *J Bioenergetics Biomembranes* **28**: 255–267.

Khan RN, Smith SK, Morrison JJ, Ashford ML. 1993. Properties of large-conductance K+ channels in human myometrium during pregnancy and labour. *Proc R Soc Lond B Biol Sci* **251**: 9–15.

Khan RN, Smith SK, Morrison JJAshfordML. 1997. Ca2+ dependence and pharmacology of large-conductance K+ channels in nonlabor and labor human uterine myocytes. *Am J Physiol* **273**: C1721–1731.

Logsdon NJ, Kang JS, Togo JA, Christian EP, Aiyar J. 1997. A novel gene hKCa4 encodes the calcium-activated potassium channel in human T lymphocytes. *J Biol Chem* **272**: 32723–32726.

Nelson MT, Cheng H, Rubart M, Santana LF, Bonev AD, Knot HJ, Lederer WJ. 1995. Relaxation of arterial smooth muscle by calcium sparks. *Science* **270**: 633–637.

Norman MR, Thompson EB. 1977. Characterization of a glucocorticoid-sensitive human lymphoid cell line. *Cancer Res* **37**: 3785–3791.

Núnêz C, Bashein AM, Brunet C, *et al.* 2000. Expression of the imprinted tumour-suppressor gene H19 is tightly regulated during normal hematopoiesis and is reduced in hematopoietic precursors of patients with polycythemia vera. *J Pathol* **190**: 61–68.

Preudhomme C, Revillion F, Merlat A, *et al.* 1999. Detection of BCR-ABL transcripts in chronic myeloid leukemia (CML) using a 'real time' quantitative RT–PCR assay. *Leukemia* **13**: 957–964.

Ramanathan K, Michael TH, Jiang GJ, Hiel H, Fuchs PA. 1999. A molecular mechanism for electrical tuning of cochlear hair cells. *Science* **283**: 215–217.

Rambhatla L, Patel B, Dhanasekaran N, Latham KE. 1995.

Copyright © 2000 John Wiley & Sons, Ltd. 

Analysis of G protein alpha subunit mRNA abundance in preimplantation mouse embryos using a rapid quantitative RT–PCR approach. *Mol Reprod Dev* **41**: 314–324.

Rappolee DA, Wang A, Mark D, Werb Z. 1989. Novel method for studying messenger-RNA phenotypes in single or small numbers of cells. *J Cell Biochem* **39**: 1–11.

Sauvageau G, Lansdorp PM, Eaves CJ, *et al*. 1994. Differential expression of homeobox genes in functionally distinct CD34(+) subpopulations of human bone-marrow cells. *Proc Natl Acad Sci USA* **91**: 12223–12227.

Song M, Zhu N, Olcese R, *et al*. 1999. Hormonal control of protein expression and mRNA levels of the MaxiK channel alpha subunit in myometrium. *FEBS Lett* **460**: 427–432.

Steuerwald N, Cohen J, Herrera RJ, Brenner CA. 1999. Analysis of gene expression in single oocytes and embryos by real-time rapid cycle fluorescence monitored RT–PCR. *Mol Hum Reprod* **5**: 1034–1039.

Trumper LH, Brady G, Bagg A, *et al*. 1993. Single-cell analysis of Hodgkin and Reed–Sternberg cells: molecular heterogeneity of gene expression and p53 mutations. *Blood* **81**: 3097–3115.

Tseng-Crank J, Godinot N, Johansen TE, *et al*. 1996. Cloning expression and distribution of a Ca2+-activated K+ channel beta-subunit from human brain. *Proc Natl Acad Sci USA* **93**: 9200–9205.

Wallner M, Meera P, Ottolia M, *et al*. 1995. Characterization of and modulation by a $\beta$-subunit of a human maxi $K_{Ca}$ channel cloned from myometrium. *Receptors Channels* **3**: 185–199.

Wang T, Brown MJ. 1999. mRNA quantification by real time TaqMan polymerase chain reaction: validation and comparison with RNase protection. *Anal Biochem* **269**: 198–201.

Yan XQ, Brady G, Iscove NN. 1993. Platelet-derived growth factor (PDGF) activates primitive hematopoietic precursors (pre-CFCmulti) by upregulating IL-1 in PDGF receptor-expressing macrophages. *J Immunol* **150**: 2440–2448.

*The*
**Genomics**
*Website at Wiley*

**www.wiley.co.uk/genomics**

The **Genomics** website at Wiley is a new and **DYNAMIC** resource for the genomics community, offering **FREE** special feature articles and new information **EACH MONTH**.

Find out more about *Comparative and Functional Genomics*, and how to view all articles published this year **FREE OF CHARGE**!

Visit the **Library** for hot books in Genomics, Bioinformatics, Molecular Genetics and more.

Click on **Primary Research** for information on all our up-to-the minute journals, including: *Genesis*, *Bioessays, Gene Function and Disease*, and the *Journal of Gene Medicine*.

Let the **Genomics** website at Wiley be your **guide** to genomics-related web sites, manufacturers and suppliers, and a calendar of conferences.

WILEY

1516

Copyright © 2000 John Wiley & Sons, Ltd. 
